# Youth vaping prevention with urban Indigenous communities of the southwest U.S.: a protocol for a randomized controlled trial

**DOI:** 10.3389/fpubh.2025.1618341

**Published:** 2025-09-03

**Authors:** Stephanie L. Ayers, Matt Ignacio, Jolyana Begay-Kroupa, Sabrina Oesterle, Scott K. Okamoto, Zoe K. Reep, Danielle June, Stephen S. Kulis

**Affiliations:** ^1^Southwest Interdisciplinary Research Center, School of Social Work, Arizona State University, Phoenix, AZ, United States; ^2^Phoenix Indian Center, Phoenix, AZ, United States; ^3^University of Hawai’i Cancer Center, Honolulu, HI, United States; ^4^Global Center for Applied Health Research, School of Social Work, Arizona State University, Phoenix, AZ, United States

**Keywords:** urban, American Indian, native American, indigenous, vaping, prevention, health equity

## Abstract

**Background:**

Indigenous adolescents have the highest rates of nicotine vaping and tend to initiate at an earlier age compared to other racial and ethnic groups. Despite this, no evidence-based intervention currently exists to prevent nicotine and cannabis vaping for Indigenous youth.

**Methods:**

This study will partner with a long-established community organization and an Indigenous Youth Advisory Board to better understand the key risk and protective factors associated with vaping among urban American Indian youth of the Southwest U.S. The study will also adapt and test through a randomized controlled trial *Living in 2 Worlds*, an empirically supported substance use prevention intervention, to specifically target nicotine and cannabis vaping in urban American Indian youth. The Indigenous Youth Advisory Board will be actively involved throughout the research process, helping to guide the study, ensure meaningful youth participation, and changes in their confidence, leadership, and collaboration will be assessed.

**Discussion:**

This study builds on the strengths of the urban Indigenous community and long-standing partnerships to address a critical need: reducing vaping disparities among Indigenous youth and their associated health impacts. By developing an evidence-based, culturally relevant, and sustainable intervention, this research aims to create lasting change and help eliminate racial and ethnic disparities in substance use.

## Introduction

The US Surgeon General declared adolescent e-cigarette vaping an epidemic in 2018 (1) due to the 13-fold increase in only 7 years (2). Indigenous adolescents have the highest prevalence and earliest initiation of nicotine vaping of all racial/ethnic groups (3, 4). Almost half of Indigenous youth report past 30-day nicotine vaping as compared to 32.7% of US youth, and Indigenous high school students are twice as likely than other students to be frequent e-cigarette users (5). While there remains a critical need to prevent nicotine and cannabis vaping among Indigenous adolescents, prevention efforts are particularly challenging due to the unique features of vaping such as ease of concealability, ability to purchase online, and perceptions of fewer adverse health consequences (6).

In the US, the Bureau of Indian Affairs officially recognizes 574 tribes (7), each with its own distinct cultural practices, languages, and traditions, reflecting the rich diversity among Indigenous peoples. This variation is also present in urban areas, where approximately 60% of Indigenous individuals live (8). Although Indigenous adolescents are exposed to influences in multiple domains (peers, family, school, and community) that can increase their risk of vaping, in the urban environment these risk factors may be amplified by the complex navigation of ethnic identity, due to geographic dispersion, cultural disconnections, and persistent discrimination (9, 10). Compared to the general urban population, urban Indigenous families experience lower socioeconomic status, employment, education, and residential stability (11). They are more likely to move frequently within urban areas and/or move between urban areas and reservation lands for short periods of time (12, 13). This pattern of mobility is not unique to urban Indigenous populations but reflects a broader pattern of geographic fluidity among many tribal communities, where individuals and families move between urban centers and tribal homelands for cultural, familial, or economic reasons (14). As a consequence, many urban Indigenous adolescents experience cultural disconnection from their ancestral homelands and have less understanding of their own tribes’ practices and traditions than tribal youth (15–18).

Because urban Indigenous residents are often scattered geographically and lack a cohesive community network, Indigenous youth often have limited social and cultural support to maintain their cultural identity, and have few opportunities to engage in traditional cultural practices (15, 19–23). Urban Indigenous youth are at increased risk of experiencing discrimination in their day to day lives. They report being victimized by racial slurs, asked if they were a “real” Indian by non-Natives, and mistaken for a race other than Indigenous (16). As a result, urban Indigenous adolescents are at risk for using substances as a coping mechanism to deal with urban life stressors tied to identity formation, as they navigate both Indigenous and non-Indigenous cultures (10, 24). However, research indicates that protective factors embedded within ethnic-racial identity (i.e., Cultural Practices and Traditions, Coping with Bias and Discrimination, Ancestral Homelands, Biculturalism, Pride, Circular Migration, and Interconnectedness with Community) can safeguard against high-risk behaviors for Indigenous youth (25–27). Preventive nicotine and cannabis vaping interventions are critically needed to target the unique and multilevel risk and protective factors of urban Indigenous youth. However, no such evidence-based intervention currently exists.

In general, vaping presents a unique challenge for prevention efforts. Unlike other substances, adolescents are able to purchase vaping products online, and for the first time in 50 years, advertisements for nicotine are directed toward youth (6). Coupled with social media content promoting vaping, adolescents are less likely to perceive the adverse health consequences of vaping (6). Moreover, motivations for adolescent vaping can also be distinct or different from smoking combustible tobacco and/or cannabis cigarettes. For example, reasons for using combustible cigarettes include coping with negative emotions, stress, or anxiety (28). However, reasons for vaping are different, as described by adolescents, who report vaping products are easier to obtain, cost less, and are more discreet than other nicotine and cannabis products (6). Additionally, adolescents may also have significant misinformation, misconceptions, and misperceptions that vaping has low health risks and is not addictive, placing them at greater harm (29). While vaping may lower health risks for adults who smoke cigarettes, vaping during adolescence and exposure to nicotine and cannabis remains an addictive choice for a developing brain (30). Research has shown that cannabis oils manufactured for vaping devices can have THC levels four times greater than that of the most potent dried cannabis plant (31). Adolescents can also be highly vulnerable to and less discerning about advertisements of vaping (32), and racial/ethnic minority adolescents are significantly more susceptible to advertising than non-Hispanic Whites (33). Despite the FDA regulating vaping advertisement targeting teens, adolescents are inundated with music videos and social media influencers showing product placements for vaping and ways to conceal vape products (34).

Indigenous adolescents may face unique vulnerabilities to vaping due to a complex intersection of historical, commercial, and cultural influences. Tobacco and e-cigarette companies have long engaged in targeted marketing toward Indigenous populations, including strategic exploitation of Tribal lands and cultural symbols, free giveaways of products, steep price reductions, highly visible charitable donations, and gaming-related promotions (35, 36). These efforts not only increased access to tobacco and vaping products in Indigenous communities but also worked to normalize their presence and usage, with some geographic variation. Data for combustible tobacco smoking suggest that Indigenous adolescents living in the Upper Great Lakes, Southeast, and Northern Plains region are more likely to have ever smoked or smoked in the past month compared to Indigenous youth from the Southwest (37). Compounding this risk is the culturally significant role of traditional tobacco, considered by many, but not all, Indigenous communities as a sacred, medicinal, and ceremonial plant that can be used to communicate with spirits, honor the dead, and promote well-beingFor example, the commercial appropriation and marketing of tobacco products using Indigenous imagery and names (e.g., *American Spirit*) blurs the line between sacred and recreational use. This blurring is further intensified when commercially produced tobacco is used in ceremonies due to limited access to traditionally grown tobacco, which typically has lower nicotine content than highly addictive commercial tobacco (38, 39). This may give adolescents the incorrect impression that using nicotine products, like vaping, is acceptable or spiritual (38). Thus, prevention interventions that have been shown efficacious in preventing other drug and alcohol use may need adaptations to specifically address vaping risks, including those unique to Indigenous youth, to have the intended impact on vaping (6).

### Framework

**Figure 1:**
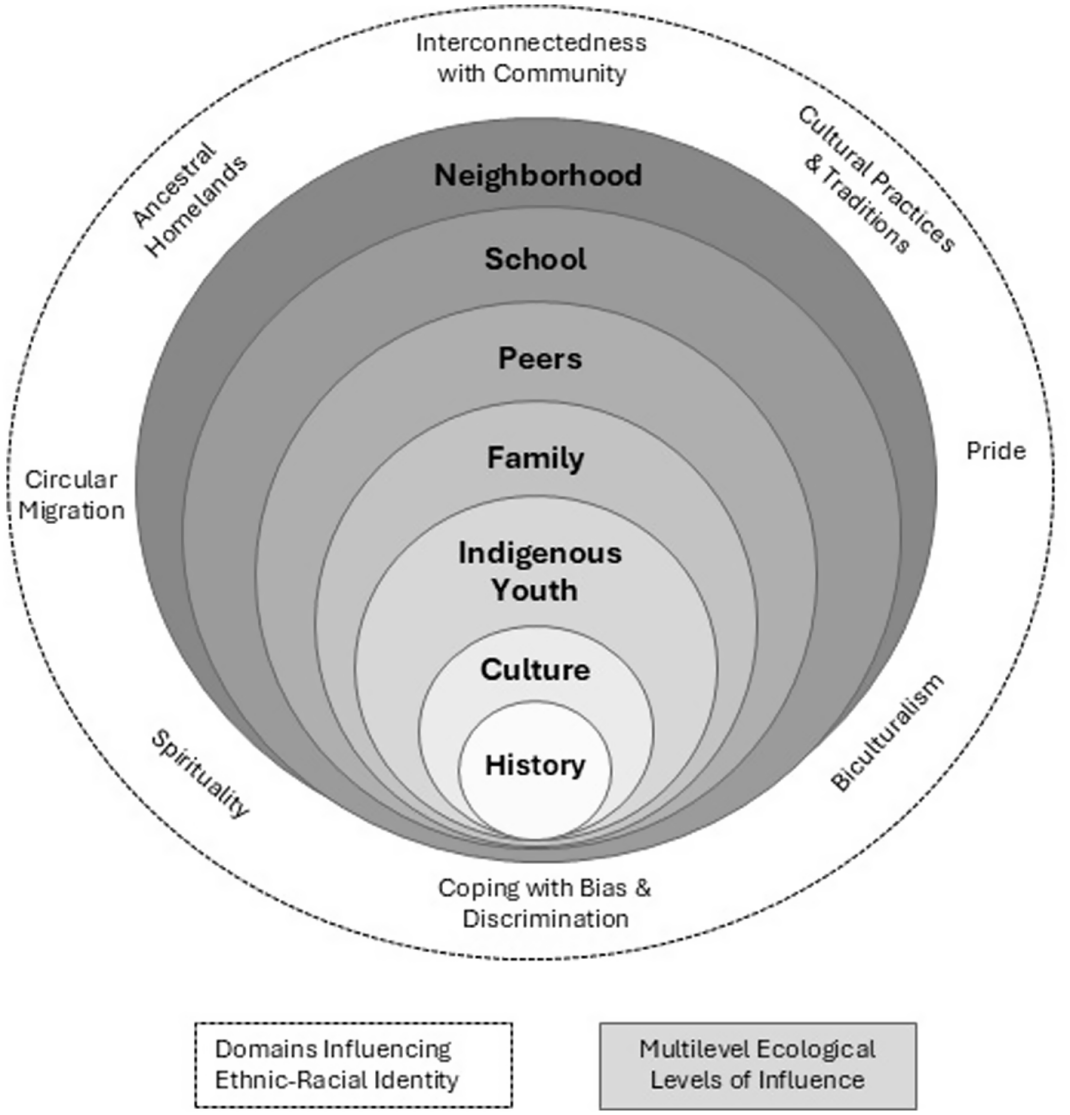
Multilevel and multidomain social ecology of urban indigenous youth.

To address this gap, we designed a study to advance the science on salient risk and protective factors for urban Indigenous adolescent nicotine and cannabis vaping and test an adapted version of *Living in 2 Worlds,* an empirically supported substance use prevention intervention, to now include nicotine and cannabis vaping prevention education. Our study is guided by the Indigenist Ecological Systems Framework (40) and NIMHD’s Minority Health and Health Disparities Research Framework, adapted for American Indian and Alaska Native Nations (41). The Indigenist Ecological Systems Framework places the Indigenous adolescent at the center, embedding history and culture withing their lived experiences to highlight the intergenerational connections between past, present, and future. This is in alignment with traditional cultural practices of American Indian cultures by placing history and culture as primary levels of influence on Indigenous youth, and requiring knowledge transmission of culturally-specific teachings and protective values from parents, elders, school, and community to improve health outcomes (42). The NIMHD Research Framework emphasizes the complex, interconnected, and multi-faceted determinants that influence health disparities among minoritized populations, and has been adapted to include factors salient for Indigenous populations (e.g., ancestral homelands) (41). As detailed in Figure 1, our study combines these frameworks, with the multilevel ecological levels of influence (Youth, Family, Peers, School, and Neighborhood) are depicted in gray, while the domains that influence urban Indigenous ethnic-racial identity (Cultural Practices and Traditions, Bias and Discrimination, Ancestral Homelands, Urban Environment, Ethnic-Racial Pride, Circular Migration, and Interconnectedness with Community) are in white.

### Substance use prevention intervention

Efficacious vaping prevention programs do not yet exist, especially for Indigenous youth. However, there are existing effective substance use prevention programs for Indigenous youth, which provide the opportunity for adaptation to include nicotine and cannabis vaping. *Living in 2 Worlds* is a multi-tribal, culturally grounded, empirically supported substance use prevention intervention for middle school-aged urban Indigenous adolescents. *Living in 2 Worlds* is a 12-lesson (see Table 1), group-based manualized curriculum delivered in 60-min sessions once a week by a trained facilitator. Skills learned in *Living in 2 Worlds* are designed to strengthen: (a) the ability to resist substance use offers; (b) risk assessments, decision-making, and problem solving around substance use; (c) knowledge of risk factors impacting substance use, including peer and family permissive substance use norms; and (d) connections with cultural values that can have a protective function. As a result, risk factors for substance use (i.e., vulnerability to substance use offers, intentions to use substances, permissive norms for substance use, perceived harmlessness of substance use) are decreased, thereby preventing and decreasing substance use (43).

**Table 1:** Living in 2 worlds lessons.

Lesson	Title	Learning objectives
1	Options and choices	Youth are introduced to *Living in 2 Worlds* and learn how to identify important factors when making a choice.
2	Living in 2 worlds	Youth explore unique aspects of their culture & recognize advantages of living in two worlds.
3	Beliefs, norms, and values	Youth identify their norms, beliefs and values, and how to align their behavior with them.
4	Avoid	Youth develop skills to avoid substance use and navigate risky situations safely.
5	Risky business	Youth identify how risk-taking can lead to harmful consequences and reflect on personal values that support making safer choices.
6	Communicating choices	Youth develop communication skills to express feelings and views assertively but respectfully, while recognizing and honoring differences in others.
7	Refuse respectfully	Youth learn how to confidently refuse to use substances or to engage in risky behaviors, through both verbal and non-verbal communication.
8	Storytelling	Youth discover how storytelling can serve as a tool for guidance and resilience in challenging or risky situations.
9	Explain	Youth build communication skills to clearly explain their reasons for choosing not to engage in substance use or other risky behaviors.
10	Help networks	Youth recognize supportive people in their lives and learn how to reach out for help when needed.
11	Leave	Youth learn how to remove themselves from situations that may lead to risky or unwanted behaviors.
12	My place in both worlds	Youth recognize the strengths in Indigenous cultures and the benefits of successfully living in both worlds.

In 2007, the Southwest Interdisciplinary Research Center at Arizona State University partnered with Phoenix Indian Center, the country’s first and oldest urban Indian non-profit established in 1947. This partnership co-created and tested *Living in 2 Worlds* in a small efficacy trial. After the conclusion of the original NIH-funded efficacy trial in 2012, Phoenix Indian Center has continued to sustain implementation of *Living in 2 Worlds.* Through these ongoing efforts, Phoenix Indian Center identified and prioritized the need to further adapt *Living in 2 Worlds* in order to address the current trends in nicotine and cannabis vaping and to strengthen ethnic-racial identity throughout the curriculum. The latter updates are necessary to ensure *Living in 2 Worlds* is inclusive of youth with diverse Arizona tribal identities, including those who have multi-racial/ethnic backgrounds, identify as nonbinary (i.e., LGBTQ/Two-Spirit), have lost connection to extended family or ancestral homelands, and face bias and discrimination from their peers, school, and community.

### Objectives

The Southwest Interdisciplinary Research Center at Arizona State University will work collaboratively with a long-standing community partner, Phoenix Indian Center, and an Indigenous Youth Advisory Board to advance knowledge on salient multilevel risk and protective factors for urban Indigenous adolescent vaping and to test an adapted version of *Living in 2 Worlds* (43) to prevent nicotine and cannabis vaping in urban Indigenous youth. The Specific Aims of the study are to: (1) Identify multilevel risk and protective factors for nicotine and cannabis vaping among urban Indigenous youth in order to adapt the *Living in 2 Worlds* intervention; (2) Test the efficacy of the adapted *Living in 2 Worlds* intervention for preventing initiation and reducing use of nicotine and cannabis vaping, decreasing key risk factors for vaping initiation, and increasing skills that protect against vaping; (2b) Identify implementation barriers and facilitators of *Living in 2 Worlds*; (2c) Explore racial/ethnic identity (American Indian only vs. Multiracial/ Multiethnic) as a moderator of the efficacy of *Living in 2 Worlds*; and (3) Advance the science for engaging youth throughout the research process.

### Qualitative inquiry

To explore the beliefs, attitudes, and behavioral norms of the multilevel risk and protective factors in Aim 1, focus groups with youth and parents/guardians will be conducted. This qualitative approach is well-suited for gaining a deeper understanding of how participants interpret, respond to, and resist nicotine and cannabis vaping within their social and cultural contexts. Focus groups offer an interactive setting where participants can express their thoughts, share personal lived experiences, and respond to others’ perspectives, often revealing insights that might not surface in individual interviews or surveys (44). This method is particularly useful for engaging historically underrepresented communities in a culturally responsive manner (45, 46). Culturally responsive focus groups foster open, authentic, strengths-based dialog that honors participants’ shared and diverse identities and helps ensure that resulting interventions are both evidence-based and culturally grounded (47).

### Quantitative hypotheses

In the Aim 2 efficacy test, we hypothesize that relative to adolescents in the comparison condition (detailed below), adolescents who participate in the adapted *Living in 2 Worlds* program will: (a) be less likely to initiate nicotine and cannabis vaping; (b) have lower vaping intentions, less permissive norms for vaping, be less vulnerable to offers, and perceive fewer benefits of vaping; (c) have better strategies for resisting vaping offers, risk assessment, decision making, and connecting with cultural values; and (d) experience similar reductions in desired alcohol, cigarettes, marijuana, and inhalants use/initiation as with the original version of *Living in 2 Worlds*. In addition, we hypothesize that youth who identify American Indian only will benefit more from the *Living in 2 Worlds* intervention than youth who identify as Multiracial/ Multiethnic. This hypothesis is based on two factors. First, youth identifying American Indian only are at greater risk for substance use, and research indicates that familial substance use has a stronger influence on cigarette use among American Indian only adolescents compare to their multiracial/multiethnic counterparts (48, 49). Second, the intervention is specifically designed to align with American Indian values, beliefs, and norms—elements that may have a stronger impact on youth who feel a closer connection to these cultural foundations. Prior research has noted that Multiracial/ Multiethnic Indigenous individuals often report weak ties to their Indigenous cultures and communities (50).

## Methods

### Study design

**Figure 2:**
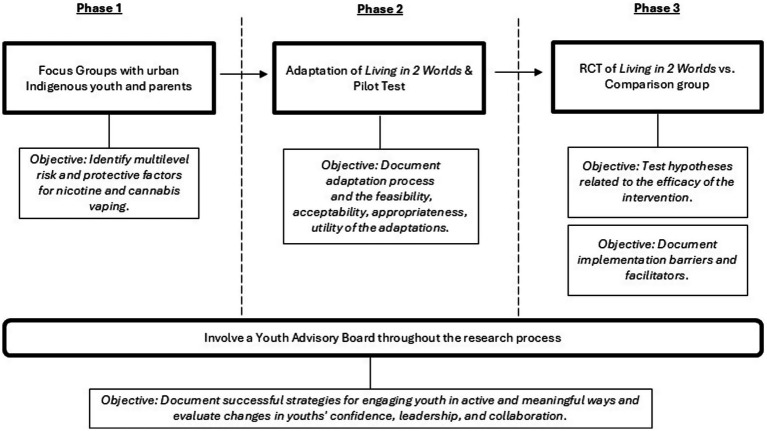
Study phases.

The three phases of this study are depicted in Figure 2. Phase 1 will advance knowledge on salient multilevel risk and protective factors for urban Indigenous adolescent nicotine and cannabis vaping. We will recruit 144 urban Indigenous adolescents and 48 parents/guardians of urban Indigenous youth to participate in one of 24 focus groups throughout urban areas in Arizona (i.e., Flagstaff, Yuma, Tucson, and Phoenix).

Phase 2 will use findings from the focus groups to adapt the *Living in 2 Worlds* intervention and pilot test with 11 urban Indigenous adolescents and the intervention facilitator in order to document the feasibility, acceptability, appropriateness, and utility of the adaptations.

Phase 3 will conduct a randomized controlled trial of the adapted *Living in 2 Worlds* intervention versus a comparison group with 360 urban Indgenous adolescents in ten public middle schools to test the hypotheses. Youth will be administered three surveys: pretest (T1 - September), an immediate post-test two months after implementation ends (T2 - January), at a time when the program effects on the prevention of short-term risks for vaping are expected to be strongest; and a follow-up posttest four months later (T3 – May).

Throughout all phases, an Indigenous Youth Advisory Board will be integrally involved to help guide the research. Successful strategies for engaging youth in active and meaningful ways in research studies will be evaluated, examining changes in youths’ confidence, leadership, and collaboration through pre-post surveys.

### Human subjects protections

Prior to study procedures, the research team will obtain written informed consent and/or assent for all phases of the study. Participants will be informed that involvement in the study is voluntary and that they are free to withdraw at any time. Research staff will be available to answer questions and administer consent/assent. Participants will receive an incentive for participating in the study: Phase 1 – $50 for parents and youth; Phase 2 – $50 for youth and facilitator; Phase 3 – $25/$35/$45 for youth T1/T2/T3 surveys and $25 for facilitators; Youth Advisory Board – $50 per in-person meeting and $25 per survey. The study protocol and all study-related documents have been approved by the Institutional Review Board at Arizona State University (STUDY00021011), titled, “Leveraging Community-Engaged Research to Co-Create Youth Vaping Prevention with Urban Indigenous Communities of the Southwest.” Phase 3 will have oversight of a Data Safety and Monitoring Board, and prior to Phase 3, the study will be registered at www.clinicaltrials.gov (ClinicalTrials.gov).

## Indigenous youth advisory board

### Participants

The Youth Advisory Board will have 12 members per year from the urban Indigenous community in metropolitan Phoenix. The Youth Advisory Board members will have varying socioeconomic backgrounds and represent diverse sex, gender, and tribal backgrounds.

### Inclusion criteria

Youth will be included if they self-identify as American Indian or Alaska Native (AI/AN; alone or in combination with another racial/ethnic group), live in an Arizona zip code located in an urban area of Phoenix, AZ, and are between the ages of 12–17 years old.

### Exclusion criteria

Youth will be excluded if they are unable or refuse to give informed assent or if their parent/guardian is unable or refuses to give informed parental permission.

### Recruitment strategies

Trained study staff will recruit participants at community events such as health fairs and pow-wows, social media, schools, youth centers, Phoenix Indian Center services, and word of mouth.

### Procedures

The goals of the Youth Advisory Board are to improve the relevance, feasibility, and sustainability of the research and to ensure authenticity of the findings. The Youth Advisory Board will be grounded in Youth Participatory Action Research (YPAR) (51) and Indigenous Methodology (52). YPAR emphasizes youth as knowledgeable contributors to research based on their lived experiences and supports their role in driving change in their communities (51), while Indigenous Methodology emphasizes community ownership, reciprocal partnerships, and shared responsibility to ensure research is meaningful, beneficial, and accountable to Indigenous communities (52).

The research team, Phoenix Indian Center, and Youth Advisory Board will jointly decide upon the processes, protocols, and products throughout the research study, including: (a) Identifying diverse recruitment and retention strategies and materials; (b) Adapting and refining the *Living in 2 Worlds* intervention materials by providing feedback, ideas, and adaptation changes to the curriculum; and (c) Translating research findings and disseminating data visualizations that build a visual story of research findings. These activities are intended to ensure urban Indigenous adolescents are empowered as co-researchers and build their strengths, capacity, and competencies to solve community problems and translate research in ways that resonate with their community (53). The Youth Advisory Board will be co-facilitated by Arizona State University and Phoenix Indian Center. The Youth Advisory Board will meet in person quarterly to help co-develop the research process and will meet online monthly to participate in learning activities and cultural connection opportunities.

Youth Advisory Board members will participate in pre/post surveys to assess changes in youths’ intrapersonal psychological empowerment and collective participation (54, 55). In the posttest, youth will be asked to reflect on their experiences, benefits, and challenges during the past year while serving on the Youth Advisory Board. Through meeting notes, we will document the ways in which youth were engaged in the process and their scientific literacy was enhanced, adding to our understandings of the impact that youth can have in adapting, implementing, testing, and disseminating youth-focused interventions.

### Methods of analysis

The Youth Advisory Board outcome evaluation will include descriptive statistics at the pre- and post-test, to examine changes in patterns of members’ responses, as well as inferential statistics to assess changes over time in outcomes of interest. The meeting notes will be assessed to ensure that key lessons are documented.

## Study phases

### Phase 1: Focus groups to identify multilevel risk and protective factors for nicotine and cannabis vaping

#### Participants

In total, 192 participants (144 youth participants; 48 parent/guardian participants) will participate in one of 24 focus groups (18 youth focus groups; 6 parent/guardian focus groups).

### Inclusion criteria

Focus group participants will be included if they self-identify as American Indian or Alaska Native (AI/AN; alone or in combination with another racial/ethnic group) and live in an Arizona zip code located in an urban area in Flagstaff, Yuma, Tucson, and Phoenix, AZ. Youth will be between the ages of 11 and 17 years old. Adults will be a parent/guardian of an AI/AN child between 11 and 17 years old and have day-to-day responsibility of parenting the child. Youth and adults may be related or unrelated to one another.

### Exclusion criteria

Individuals will be excluded if they are unable or refuse to give informed consent/assent or the youth’s parent/guardian is unable or refuses to give informed parental permission.

### Recruitment strategies

Phoenix Indian Center, with support from Arizona State University, will lead the recruitment efforts drawing on their extensive experience in recruiting participants for research studies, as well as, on their trusted relationships with urban Indigenous families. Recruitment strategies, such as through Phoenix Indian Center’s services, schools, youth centers, health fairs, pow-wows, social media, and word of mouth will be developed and expanded through the Youth Advisory Board.

### Procedures

In order to ensure that diverse adolescent perspectives from across Arizona are represented, youth focus groups will be organized around three categories: (1) non-users – individuals who have never vaped nicotine nor cannabis; (2) contemplators or experimenters – individuals who have tried either nicotine or cannabis vaping or considered trying it, but do not vape nicotine or cannabis regularly; and (3) regular users – individuals who vape either nicotine or cannabis frequently, such as several times per week or daily. In addition, adolescents will also have the option to join the group that aligns best with their sex or gender identity, based on where they feel most comfortable.

Focus groups will occur in-person with 6–8 participants per group, last 90 min, and be audio recorded. Focus groups will be led by two trained facilitators using a semi-structured moderators’ guide that will facilitate discussions, and notes will be taken in order to enhance the accuracy of transcribed data. At the start of each focus group, ground rules will be established to promote a respectful and inclusive environment—at minimum, encouraging participants to speak one at a time, listen actively, and use respectful language to ensure that all voices are heard and valued. Examples of discussion topics include the risks and benefits of nicotine / cannabis vaping, culturally- and community-specific protective factors for nicotine / cannabis vaping, the influence of parents, peers, and teachers on nicotine / cannabis vaping, how neighborhoods impact nicotine / cannabis vaping, the impact of ethnic-racial identity on nicotine / cannabis vaping, and strategies to counter the negative effects of discrimination that help prevent adolescents from nicotine / cannabis vaping.

### Method of analysis

We will use three strategies during the data analysis process to reinforce scientific rigor. First, audio-recordings will be transcribed and verified for accuracy prior to qualitative analysis. All identifying information will be removed. Second, research staff will review focus group recordings on an ongoing basis to identify additional areas of inquiry for subsequent focus groups as needed. Third, we will use data triangulation to compare and identify congruence (or discrepancies) between nicotine and cannabis vaping, as well as between youth and parents (56). Three Indigenous researchers with diverse lived experiences will code and analyze the data.

An inductive thematic analysis approach will be used in three linked, iterative stages: (1) *Data reduction*. De-identified transcripts will be read independently by two coders to identify segments of text that are important or meaningful. The coding process will begin with identifying and categorizing text related to nicotine vaping, including behaviors, risk and protective factors, resistance strategies, and cultural considerations. This same process will then be applied to cannabis vaping, with coders identifying relevant content across the same domains to ensure consistency and comprehensive analysis. Any additional codes will be organized into existing or new categories supported with operational definitions (57). To ensure findings are grounded in the dataset, categories and codes will be compared to original raw data to ensure themes are mutually exclusive, yet broad enough to capture a set of ideas based on direct quotes. Intercoder agreement will be established by two reviewers, and any discrepancies in codes, categories, or definitions will be resolved by a third reviewer to ensure consensus among researchers. (2) *Data display*. We will organize codes into a visual display using the Indigenous Ecological framework to underscore how risky behaviors and environments, as well as resistance strategies and protective factors for vaping among youth are structurally and socially dependent on the places in which they occur (58). We will analyze the findings for areas of convergence between nicotine and cannabis vaping to identify common themes and patterns that can be integrated into the adapted *Living in 2 Worlds* curriculum. In parallel, we will examine points of divergence to determine where distinct aspects of nicotine or cannabis vaping may require separate attention, ensuring that the adaptation includes content specific to each substance where appropriate. The analysis will follow the same approach previously applied to adapt curricula for alcohol, cigarettes, marijuana, and inhalants in *Living in 2 Worlds* (59) and its predecessor, *keepin’ it REAL (kiR)* (60), an evidence-based, universal school-based substance use prevention intervention. (3) *Member Checking*. The codes, categories, and definitions will be reviewed by Phoenix Indian Center staff and the Youth Advisory Board for accuracy.

### Phase 2: Intervention adaptation and pilot test

#### Adaptation

The adaptation process will be guided by the Model for Adaptation Design and Impact, a framework that systematically directs intervention modifications while emphasizing the causal pathways through which adaptation characteristics influence implementation and intervention outcomes (61). This systematic and prospective decision-making approach helps to ensure that adaptations are aligned with the core elements of the intervention, made with a clear goal, and produce positive impacts on implementation and intervention outcomes (61). In collaboration with Phoenix Indian Center and the Youth Advisory Board, the adaptation process will embed multilevel risk and protective factors for nicotine and cannabis vaping in ways that resonate with youth and increase community ownership of *Living in 2 Worlds* (62). Focus group findings will be presented to the research team, Phoenix Indian Center, and the Youth Advisory Board to gather feedback, ideas, and potential adaptations to *Living in 2 Worlds*. We will use various activities (e.g., brainstorming, group discussion) and visual tools (e.g., logic models) to create a roadmap that will inform the adaptations. In addition, we will partner with an Indigenous curriculum designer to ensure Indigenous ways of learning (i.e., circular and wholistic rather than linear and sequential), along with artistic and visual expressions of culture are integrated into *Living in 2 Worlds*. We anticipate adaptations to include adding elements, like examples and activities; substituting elements, like replacing a lesson, activity, or video that addresses nicotine vaping rather than cigarette smoking; and/or deleting elementsno longer temporally appropriate, like “MySpace.” Futhermore, adaptations will also incorporate scenarios that reflect real-life situations in which adolescents encounter risks related to nicotine and cannabis vaping separately, along with effective resistance strategies. These scenarios will be drawn from key themes identified through the focus group analysis. Moreover, some activities may be adapted to specifically address either nicotine or cannabis vaping, depending on the unique focus group findings related to each substance. In addition, given the Phoenix Indian Center’s extensive experience in implementing *Living in 2 Worlds* for the past 13 years, their knowledge and expertise of the pacing of the curriculum, clarity and consistency of materials, usability and alignment of the manual will also be considered during the adaptation process.

### Pilot participants

We will pilot test the adapted version of the *Living in 2 Worlds* intervention in one group of 11 urban Indigenous adolescents recruited through Phoenix Indian Center.

### Inclusion criteria

Youth will be included if they self-identify as American Indian or Alaska Native (AI/AN; alone or in combination with another racial/ethnic group), live in an Arizona zip code located in an urban area of Phoenix, AZ, and are between the ages of 11–14 years old.

### Exclusion criteria

Youth will be excluded if they are unable or refuse to give informed assent or if their parent/guardian is unable or refuses to give informed parental permission.

### Pilot evaluation

The pilot test will serve to gather feedback from youth and the group facilitator around the feasibility, acceptability, appropriateness, of the adaptations. At the conclusion of the pilot implementation, youth will participate in a focus group to gauge if the adapted version of *Living in 2 Worlds* was realistic, believable, fun, provided helpful information on both nicotine and cannabis vaping, and if any changes were needed. The pilot facilitator will provide feedback through an in-depth interview on the strengths and challenges of implementing the intervention components, recommendations for changes to the intervention, and feedback on implementation. We will use questions from the Acceptability of Intervention Measures (AIM), Intervention Appropriateness Measure (IAM), and Feasibility of Intervention Measures (FIM) (63) to guide the in-depth interview. Acceptability will appraise if *Living in 2 Worlds* is enjoyable and satisfactory. Appropriateness will assess the perceived fit of *Living in 2 Worlds*, and feasibility will gauge if *Living in 2 Worlds* can be successfully used and implemented in the schools. These data will be analyzed using the same qualitative methods applied to the Phase 1 focus groups. Findings will be used to inform final adaptations and develop the finalized version of the adapted curriculum.

### Method of analysis

Throughout the entire adaptation process, we will create a matrix of change objectives that will document: (a) what was modified; (b) the type of modification – context or content level; (c) the reasons and rationale for the modification; (d) the outcome, objective, and theoretical foundation targeted by the change, and (e) the data which those changes were derived (64). It is critically important to document the adaptation process, as modifications to evidence-based programs have not been well-documented, understood, or consistently undertaken. Specifying the adaptation processes will support reproducibility and enhance implementation and sustainment (65).

### Phase 3: Randomized controlled trial (RCT) of the vaping adaptation of living in 2 worlds

#### Randomization of schools

Ten middle schools in the Phoenix metropolitan area that have a Native American Education Program will be invited to participate in the study over the course of two cohorts (one cohort per school year). The Native American Education Program is administered by the Office of Indian Education at the Arizona Department of Education with the goal of supporting academic and culture needs of Native American students in Arizona. Schools will be randomly assigned into either: (1) *Living in 2 Worlds* Intervention (5 schools; 180 adolescents; 36 per school) or (2) Comparison group (5 schools; 180 adolescents; 36 per school).

### Inclusion criteria

Participants will be included if they: (a) are an adolescent in 6th – 8th grade (generally 11–14 years old); (b) self-identify as American Indian/Alaska Native alone or in combination with another racial group or a Hispanic ethnic group; and (c) attend one of the urban schools participating in the trial.

### Exclusion criteria

Youth will be excluded if they are unable or refuse to give informed assent or if their parent/guardian is unable or refuses to give informed parental permission.

### Recruitment

Through the auspices of the Native American Education Program, Indigenous students enrolled in the randomized school will be invited to participate in the RCT. A letter will be sent home to obtain parental permission, and the study will be conducted during regular school hours throughout the school year.

### Intervention group

The *Living in 2 Worlds* intervention groups will be facilitated by Phoenix Indian Center. During the Fall semester, youth will meet at school for 12 weeks, one hour per week, at convenient times (e.g., during lunch period). Urban Indigenous, trained facilitators from Phoenix Indian Center will deliver the *Living in 2 Worlds* curriculum.

### Comparison group

The comparison group will only complete surveys during the academic year on the same schedule as the *Living in 2 Worlds* intervention group. After RCT data collection is complete, the comparison group will have the opportunity to participate in *Living in 2 Worlds* at no cost during a summer camp implementation to ensure all adolescents have access to the curriculum.

### Survey data collection

All youth will complete a 30-min self-administered pretest questionnaire, administered electronically via a tablet, one week prior to Living in 2 Worlds beginning (T1 - September), an immediate post-test two months after implementation ends (T2 - January), at a time when the program effects on the prevention of short-term risks for vaping are expected to be strongest, and a follow-up posttest four months later (T3 – May) to assess longer-term efficacy of the intervention at the end of the school year.

### Study measures

#### Vaping behaviors

Key vaping outcomes are modeled after the Monitoring the Future survey for youths’ self-reports of recent (last 30 days) and lifetime vaping behaviors (66). Vaping nicotine will be defined as “vaping nicotine (using a JUUL, e-cigarette, e-pen)” (66) and vaping cannabis will be defined as “vaping marijuana or cannabis (e.g., using cannabis oils or liquids, dried herbs, or a cannabis concentrate, like wax, shatter, or budder)” (67). Separate but parallel questions will assess nicotine and cannabis vaping. These items include: During the last 30 days [lifetime] have you vaped nicotine [cannabis]? On how many days (if any) during the last 30 days [lifetime] have you vaped nicotine [cannabis]? Did you first start vaping nicotine [cannabis] in the last 30 days? There is sufficient evidence of the validity of self-reports and for comparing self-reports over time (68).

#### Risk factors for vaping [scales]

Measures of key risk factors for vaping will be drawn from the Monitoring the Future survey (66) and our prior *Living in 2 Worlds* study (43). Separate but parallel questions will assess nicotine and cannabis vaping. *Intentions to vape* will be assessed by asking adolescents, “If you had the chance this weekend, would you vape nicotine [cannabis]?” *Permissive norms* for vaping will be assessed for the adolescent, close friends, parents, and grandparents by asking: “Is it OK for someone your age to vape nicotine [cannabis]?; “How many of your friends would you estimate vape nicotine [cannabis];” and “How angry would your parents [grandparents] be if they found out you vaped nicotine [cannabis]?” *Vulnerability to offers* will gauge the extent to which the adolescent is confident they would decline an offer to vape from a family member, close friend, and a school peer. *Perceived benefits* of vaping will assess perceptions of the positive consequences of vaping, (e.g., whether vaping reduces nervousness or looks cool). *Perceived harms* of vaping will ask, “How much do you think people risk harming themselves (physically or in other ways) if they vape an e-liquid with nicotine [cannabis] occasionally,” and “…vape an e-liquid with nicotine [cannabis] regularly?

#### Skills acquired in *Living in 2 Worlds* [scales]

Based on our prior studies (43, 69), the *drug resistance strategies* measures will assess adolescent’s responses to vaping offers: the likelihood that they would turn down an offer (refuse); give an explanation or excuse (explain); stay away from situations (avoid); or leave the situation (leave). *Risk assessment* will gauge ways that the adolescent evaluates the risk of accepting the offer to vape including thinking about what it would do to their health, and the possibility a parent or elder might find out. Measures of *Decision-making/Problem-solving skills* will include questions concerning how the adolescent solves important problems like letting someone else decide or doing what others do. *Connections with cultural values* will be assessed through three validated instruments: American Indian ethnic identity (70), connections to American Indian spirituality (71), and involvement with American Indian cultural traditions (72).

#### Additional substance use

Youths’ self-reports of recent (last 30 days) frequency and amount of alcohol, cigarettes, marijuana (excluding cannabis vaping), and inhalants will be measured with items from the prior *Living in 2 Worlds* study (43).

### Statistical data analysis plans

Descriptive statistics will be assessed to identify data entry errors, outliers, and variable distributions. Scales with Chronbach’s *α* coefficients of at least 0.70 will be deemed sufficiently reliable to be included in further analysis. To test the hypotheses for nicotine and cannabis vaping, we will employ intent-to-treat models, separately for each substance. To examine change in outcomes between T1, T2, and T3, we will use general linear models and latent change models. Latent change models are appropriate for analyzing repeated-measures because these models adjust for measurement error, reduce estimate bias, and simultaneously assess changes within and between intervention and comparison groups, the timing of group differences, and their magnitude and direction. They can model a variety of trajectories of change and assess T1 to T2 changes separately from T2 to T3, indicating whether short-term changes in outcomes continue their trajectory, plateau or reverse direction (73). We will assess global model fit using the model chi-square; normed chi-square; comparative fix index; and root mean square error of approximation, following established cutoff criteria (74).

For all models, we will use the appropriate link function based on the level of measurement of the dependent variable. All models will control for demographic characteristics of participants where baseline equivalence between the treatment and comparison group are not achieved (e.g., sex, age, usual grades in school, two-parent household, receipt of free lunch at school), as well as control for school to adjust standard errors for the nested nature of the data (students nested in schools). Because of the small number of schools, multi-level models cannot be employed as they could lead to slightly inflated Type 1 error rates (75). We will adjust for missing data due to any attrition by employing full-information maximum likelihood (FIML) estimation. To determine if the null hypotheses should be rejected, effect sizes and their confidence intervals will assess the precision and substantive significance, and exact *p*-values will assess Type I error. We will also test if the adapted *Living in 2 Worlds* maintains effect sizes for other substance use seen in the original efficacy trial of *Living in 2 Worlds* (43). Based on a prior meta-analysis of universal substance use prevention programs for adolescents in grades 6 and 7, the overall average effect sizes were: *d* = 0.14 for smoking, *d* = 0.10 for alcohol use, and *d* = 0.14 for drug use (76). Based on this, we selected a non-inferiority margin of *d* = 0.14, which reflects approximately 50% of the effect size established in the original efficacy trial of *Living in 2 Worlds*.

We will test for moderation through interaction terms in general linear models (77) and multigroup analyses in latent change models (78). Multigroup analysis simultaneously tests separate models for each group. To test if parameter estimates of outcomes in the intervention conditions are equal across groups, we will use the chi-square difference test and compare model fit using model constraint methods for the main intervention effect path. A significant increase in chi-square between the two models (constrained-*M*_0_ vs. free-*M*_1_) indicates significant intervention effects between groups.

Sensitivity analyses will be conducted to test if the direction/magnitude of results differ based upon the sex of the youth. Although the study is powered to detect differences greater than *d* = 0.31, patterns of differences can be noted for future investigation. In addition, to respond to Phoenix Indian Center’s request to evaluate the summer camp version of *Living in 2 Worlds*, we will use a one group within-group pre-posttest design with paired t-tests (for continuous variables) or a McNemar’s chi-square test (for dichotomous variables) to examine the extent of change in vaping and associated outcomes of interest from the T3 RCT survey, which will serve as the summer camp pretest, to a post-test at the conclusion of summer camp.

#### Sample size calculations

Sample size calculations for the main effects of the intervention were estimated from the average effect of cigarette and marijuana use in the original *Living in 2 Worlds* efficacy trial (*d* = 0.31) and through a systematic review of school-based e-cigarette preventive interventions (*d* = 0.47). With a sample size of 132 adolescents, an effect size *of d* = 0.31 can be detected with 0.80 power for a significant intervention difference assuming a Type I error rate of *d* = 0.31, *α* = 0.05, given a pretest R^2^ = 0.67 and a 20% attrition rate. Effect sizes for the moderation analysis were estimated comparing alcohol use of urban Indigenous students who self-identified as American Indian only to students who self-identified as multiracial/ multiethnic (OR = 1.41; *d* = 0.19) (48). Assumingα = 0.05, a pretest R^2^ = 0.67, and a 20% attrition rate, we will have 80% power to detect *d* = 0.19, for statistically significant moderated intervention effects with 360 urban Indigenous adolescents ages 11–14.

#### Implementation barriers and facilitators

Throughout the RCT implementation, outcomes of acceptability, appropriateness, utility, and fidelity of *Living in 2 Worlds* will be documented. The group facilitators who deliver *Living in 2 Worlds* will provide feedback through self-administered surveys using the Acceptability of Intervention Measures (AIM), Intervention Appropriateness Measure (IAM), and Feasibility of Intervention Measures (FIM) (63). With Phoenix Indian Center, we will assess key resource constraints (e.g., time, money, personnel, technology, and barriers related to school recruitment and program adoption) that may limit the sustainability of the intervention. Implementation fidelity will be assessed by the research team and includes adherence to program guidelines and the quantity /duration of sessions, the quality of program delivery, and the extent of participant engagement (79). Data regarding implementation fidelity will be collected by observing the delivery of *Living in 2 Worlds* curriculum lessons. Trained graduate students will conduct lesson observations at three different time points. The first observation will occur within the earliest weeks of the curriculum, and two subsequent observations will take place during core concept lessons (lessons 7 and 9). Implementation fidelity will be assessed via an instrument used successfully for fidelity measurement in the prior *Living in 2 Worlds* trial (43). Data will be analyzed to examine implementation barriers and facilitators *Living in 2 Worlds* through descriptive statistics of means and standard deviations.

## Discussion

Given the existing evidence that *Living in 2 Worlds* can reduce other substance use, building new vaping prevention into *Living in 2 Worlds* is an efficient and effective approach to address this community-driven priority and reduce health disparities for urban Indigenous youth, a population at high risk but underrepresented in prevention programs. This proposed project extends the rigor of prior research and addresses a critical community-driven need. Although much is known about the prevalence and demographic characteristics of youth vaping in general and how ethnic-racial identity can protect against substance use on tribal lands, prior research has not described multilevel risk and protective factors associated with vaping for urban Indigenous youth (80).

Embedding salient risk and protective factors within the multilevel ecological systems (Family, Peers, School, and Neighborhood) and domains influencing ethnic-racial identity (Cultural Practices and Traditions, Coping with Bias and Discrimination, Ancestral Homelands, Biculturalism, Ethnic-Racial Pride, Circular Migration, and Community Connectedness) into a culturally grounded substance use prevention intervention will reduce vaping disparities among urban Indigenous youth and address the critical need for culturally grounded approaches that places culture at the center of preventive messages. Because a large majority of Indigenous families live in cities, having an evidence-based intervention that is acceptable, appropriate, feasible, and sustainable will increase the likelihood of real-world impact in eliminating racial disparities in substance use. This study will advance understandings of how to design and implement strength-based interventions for Indigenous adolescents. Understanding factors that contribute to successful implementation will ensure successful scale-up and sustainability (81).

Our community engagement process with Phoenix Indian Center and the Youth Advisory Board will enable us to draw conclusions that are robust and accurate due to the integration of various viewpoints and the lived experiences, recommendations, and solutions from members of a community experiencing health disparities in vaping (82). This collaborative approach enhances the relevance, acceptability, and cultural fit of the intervention, increasing the likelihood of real-world impact in eliminating health disparities in substance use. In addition, engaging the community throughout the research process will strengthen research accountability, ensure solutions are community-driven, and support sustainability over time.

## Conclusion

Drawing on the tremendous strengths within the urban Indigenous community and grounded in a long-standing community partnership, this study seeks to address a critical, community-identified need to prevent and reduce nicotine and cannabis vaping disparities among urban Indigenous youth and mitigate associated long-term health disparities. By adapting and improving upon the *Living in 2 Worlds* intervention—an empirically supported program with demonstrated cultural relevance—this study aims to deliver an approach that is acceptable, appropriate, and sustainable within real-world settings. Specifically, this study is guided by three primary aims: (1) to identify multilevel risk and protective factors related to nicotine and cannabis vaping in order to inform the adaptation of *Living in 2 Worlds*; (2) to test the efficacy of the adapted intervention in preventing initiation and reducing use of nicotine and cannabis vaping, while decreasing key risk factors and strengthening protective skills among youth; and (3) to advance the science of youth-engaged research and centering youth voices in all phases of the research process. Findings from this study will not only inform scalable, culturally grounded vaping prevention strategies, but also offer a framework for adapting and implementing similar interventions in other underserved communities and for expanding *Living in 2 Worlds* to other diverse real-world settings, such as to community-based organizations. Additionally, continued engagement of Indigenous youth as research partners will remain a priority to ensure relevance, empowerment, and sustained impact in addressing substance use disparities.
